# Characterization of susceptibility patterns and adaptability of the newly emerged *Candida auris*

**DOI:** 10.1007/s10123-024-00563-1

**Published:** 2024-08-07

**Authors:** Matlou D. Semenya, Adebowale E. Aladejana, Sizwe I. Ndlovu

**Affiliations:** Department of Biotechnology and Food Technology, Faculty of Science, University of Johannesburg, Johannesburg, 2028 South Africa

**Keywords:** *Candida auris*, Resistance, Adaptive features, Halotolerance, Thermotolerance, Virulence traits

## Abstract

The emergence of *Candida auris* has caused a major concern in the public health worldwide. This novel fungus is characterized by its multidrug resistance profile, ability to thrive in harsh and stressful conditions, as well as high temperatures and salt concentrations, persistence on hospital surfaces, causing nosocomial infections and outbreaks, and unique fitness properties. Here, we study the antifungal susceptibility patterns, thermotolerance, and halotolerance of 15 putative *C. auris* clinical isolates from Inkosi Albert Academic Hospital, Durban, South Africa. Five of the *C. auris* isolates showed resistance to all three antifungals (fluconazole, amphotericin B, and micafungin) and were selected for characterization of their adaptability mechanisms. Four of the tested multidrug-resistant *C. auris* isolates (*C. auris* strain F25, *C. auris* strain F276, *C. auris* F283, and *C. auris* M153) showed good growth when exposed to high temperature (42 °C) and salinity (10% NaCl) conditions whereas one isolate (*C. auris* F65) showed moderate growth under these conditions. *Candida parapsilosis* showed poor growth whereas *C. albicans* no growth under these conditions. The five *C. auris* strains were positive for all the adaptive features.

**Supplementary Information:**

The online version contains supplementary material available at 10.1007/s10123-024-00563-1.

## Introduction

*Candida auris* is an emerging fungal pathogen that has recently caused major fungal outbreaks and is considered a major global health problem due to its vast inherent and acquired antifungal drug resistance (Sikora et al. 2023). Scientists have observed an immediate rise in infection incidents and epidemics in medical facilities worldwide since its initial discovery in 2009 at Tokyo Geriatric Metropolitan Hospital (Vila et al. 2020). Since its discovery in Japan, more than 40 countries including South Africa have documented *C. auris* infections, with fatality rates ranging from 30 to 72% (Chowdhary et al. 2023). *Candida auris* was the third most prevalent cause of candidemia in South Africa in 2016–2017, accounting for one out of every ten cases of candidemia in healthcare centres, and the second most common cause of candidemia globally during that period (Naicker et al. 2023).

*Candida auris* appeared simultaneously and independently in four geographical locations with different clades including the South Asia clade (clade I), East Asia clade (clade II), Africa clade (clade III), and South America clade (clade IV) (Lockhart et al. 2017). Chow et al. (2018) identified a probable fifth clade that differs from the others by more than 200,000 single-nucleotide polymorphisms (SNPs) in an Iranian patient with no travelling history outside Iran (Chow et al. 2018). Due to its phenotypic similarities to *C. haemulonii*, *C. famata*, *C. sake*, *Saccharomyces cerevisiae*, and *Rhodotorula glutinis*, it is speculated that this fungus may have also emerged in other nations but was misidentified (Lone and Ahmad 2019). Although the *C. auris* clades differ genetically by thousands of SNPs, independent clonal expansions often occur within each clade during an outbreak (Ross and Lorenz 2020). Given that these *C. auris* clades differ genetically and have varied levels of antifungal drug resistance, it is likely that they will keep on phenotypically diverging in the future (Rhodes and Fisher 2019).

The major barriers to *C. auris* control include common misidentification as *C. haemulonii* or other related yeast species by diagnostic platforms (such as VITEK 2 YST, API 20C, Microscan, BD Phoenix yeast identification system, and Auxacolor) which are commonly used diagnostic systems clinical laboratories. Also, a lack of resistance breakpoints to antifungals, and a greater proclivity to contaminate healthcare settings, resulting in the spread of clonal *C. auris* isolates (Kordalewska and Perlin 2019). As a result, timely and precise laboratory identification is important for early control measures and timeous treatment to minimize the spread of this multidrug-resistant fungus in healthcare facilities (Kordalewska and Perlin 2022). More advanced technologies, like matrix-assisted laser desorption/ionization time-of-flight mass spectrometry (MALDI TOF MS), molecular identification based on sequencing the internal transcribed spacer (ITS), and VITEK 2 with a software version 8.01, are widely accepted as the gold standards for accurate *C. auris* identification (Lockhart et al. 2022).

Microbial adaptability is an organism’s ability to adjust to changing environmental conditions and survive in a variety of environments. *Candida auris* has several adaptive features, including genetic variability, biofilm formation, environmental tolerance (e.g., thermo- and halotolerance), and antibiotic resistance (Kogut 1980; Wani et al. 2022). Temperature, salinity, and pH can all have an impact on a pathogen’s ability to cause disease (Brumfield et al. 2021; Hellberg and Chu 2016; Bernardo-Cravo et al. 2020).

Virulence is a microorganism’s ability to infect and cause disease in its host organism (Khan et al. 2010). It is influenced by a variety of factors that increase the fungi’s ability to colonize and harm the host (Pontes et al. 2020). These characteristics include the ability to adhere to host cells and resist physical removal, invade host cells, compete for nutrients, resist antibiotics, evade innate immune defences such as phagocytosis and complement, and evade adaptive immune responses (Hernández-Chávez et al. 2017). Effectors, mycotoxins, cell wall–degrading enzymes, and organic acids are examples of virulence factors that pathogens use to control the host’s living tissues (Kumar et al. 2024). The strategies used by fungi frequently mask the host’s defence mechanism, resulting in successful infection (Kumar et al. 2024). The primary distinction between *Candida* species (such as *C. auris* and *C. albicans*) that produce virulence factors and those that do not (including *C*. *intermedia* and *C. orthopsilosis*) is their ability to invade and damage host tissues (O'Donnell et al. 2015). Virulence factors, along with microbial adaptive features, contribute to pathogenicity by increasing not only the infectivity of pathogenic fungi but also by exacerbating antimicrobial resistance, thus limiting treatment options (Ciurea et al. 2020). Multiple connections and interactions between these factors are a distinguishing feature that plays a significant role in the establishment of *Candida* infection and the failure of therapeutic approaches to treat candidiasis (Mba and Nweze 2020).

*Candida auris* often demonstrates intrinsic resistance to fluconazole and varied resistance profile to other azoles, amphotericin B, and echinocandins (Dahiya et al. 2020). *Candida* species generally use three antifungal resistance mechanisms which include the mutational changes in the antifungal target, preventing the effectiveness of an antifungal agent, mutations in the transcriptional elements of the target resulting in overexpression or changes in ploidy, and increased expression of efflux pumps that pump out the antifungal agent from the cell (Lockhart 2019). Antifungal resistance presents a significant threat in clinical settings given the limited arsenal of systematically available antifungal agents (Wiederhold 2017). As a result, a better comprehension of the susceptibility patterns of emerging fungal pathogens and their mechanisms of resistance is critical for timely and effective treatment of patients with invasive fungal infections (Lamoth et al. 2022).

*Candida auris*, like other *Candida* species, possesses and employs several features that enable fungal penetration and survival in its human host and contribute to the continued progression of infection (Watkins et al. 2022). These include adherence genes allowing for *C. auris* to adhere to surfaces, formation of biofilms that are hard to penetrate with antifungal agents, production of extracellular enzymes such as phospholipase, and proteinase that contribute to the penetration of the human host cells, drug resistance, and pathogenicity (Pokhrel et al. 2022). *Candida auris* encounters the human host primarily through contact with contaminated surfaces or medical equipment, leading to colonization in various anatomical sites (Thatchanamoorthy et al. 2022; Fernandes et al. 2022). Its ability to adhere to and invade epithelial surfaces, coupled with its resistance to desiccation, facilitates its survival and transmission between individuals (Fernandes et al. 2022). Once within the host, either through invasive medical procedures, breaches in the skin or mucous membranes due to wounds or medical devices, or dissemination from other colonized or infected sites, *C. auris* can colonize diverse bodily regions, including the skin, mucous membranes, respiratory tract, and bloodstream, with a proclivity for causing invasive infections, especially in immunocompromised patients (Ahmad and Alfouzan 2021; Kramer et al. 2022). Moreover, its capacity to form biofilms on medical devices further complicates treatment and eradication efforts (Ahmad and Alfouzan 2021; Cortegiani et al. 2018). Therefore, comprehending the mechanisms underlying *C. auris* colonization and dissemination is crucial for the development of new antifungal drugs.

*Candida auris* differs from other *Candida* species and even its close relatives in that they can grow at temperatures as high as 42 °C and 10% salinity (Jackson et al. 2019). Recently, *C. auris* was isolated from a salt march reservoir where it may have adapted to a warm and highly saline environment (Nnadi and Carter 2021). The ability of *C. auris* to survive under elevated temperatures and saline environments compared to other *Candida* species is widely accepted to be a function of adaptation to climatic variations (Casadevall et al. 2019). Mammalian bodies are endothermic with a high basal body temperature compared to the surrounding environments (Garcia-Bustos et al. 2023). This often serves as a barrier limiting infection by most environmental strains (Du et al. 2020). The narrowing of temperatures between the environment and the human body will allow more emerging fungal diseases in the future as potential pathogenic environmental fungal strains gain access and thrive at human body temperature (Chakrabarti and Sood 2021). Thermotolerance and halotolerance are advantageous fitness factors to thrive on skin, particularly in the axilla and groin, which are subject to higher temperatures as well as high salinity (Billamboz et al. 2021).

The adaptability of *C. auris* is a major concern in healthcare settings because it increases the risk of nosocomial (hospital-acquired) infections and complicates treatment. Understanding the mechanisms underlying *C. auris*’ adaptability is critical for developing effective prevention and control strategies, as well as novel antifungal agents and therapeutic approaches. *Candida albicans*, *Candida parapsilosis*, and *C. auris* are members of the CTG clade within the *Candida* genus, which has a distinct genetic code in which the CTG codon is translated as serine rather than leucine (Gómez-Gaviria et al. 2023). While *C. albicans* and *C. parapsilosis* are well-known and extensively studied pathogenic yeasts (Kabir et al. 2012; de Aguiar Cordeiro et al. 2017), *C. auris* is a relatively new species that has emerged as a significant healthcare-associated pathogen, owing to its ability to cause difficult-to-treat infections and susceptibility to nosocomial transmission (Chowdhary et al. 2017; Cristina et al. 2023). *Candida albicans* and *C. parapsilosis* were used as controls in this study because they share taxonomic and genetic similarities with *C. auris*, allowing for comparative analyses that can shed light on *C. auris*’ unique pathogenic mechanisms, drug resistance profiles, and epidemiological characteristics, as well as providing a framework for developing effective diagnostic, preventive, and therapeutic strategies.

This study, therefore, aims to assess the susceptibility patterns of *C. auris* against antifungal drugs from three main antifungal classes. We also intend to investigate the adaptive strategies of *C. auris* with a multidrug-resistance phenotype in order to predict the mechanisms by which this pathogen enters and thrives in its human hosts, as well as its adaptation to a variety of environmental factors, all of which contribute to increased infections and high mortality rates.

## Materials and methods

### *Candida* auris isolate collection

Fifteen putative clinical *C. auris* isolates and two *Candida* control strains (*C. albicans* ATCC 90028 and *C. parapsilosis* ATCC 22019) used in this study were obtained from the National Health Laboratory Services (NHLS) at Inkosi Albert Luthuli Central Hospital in Durban, KwaZulu Natal. The isolates were cultivated on Sabouraud’s dextrose agar (SDA, Neogen, UK) supplemented with 50 µg/mL chloramphenicol (Sigma-Aldrich, USA) to prevent bacterial growth. The agar plates were then incubated for 48 h at 30 °C.

### Molecular identification of C. auris isolates

The genomic DNA of the *C. auris* isolates and *Candida* control strains was isolated following the method from Harju et al. (2004). This was followed by amplification and sequencing of the internal transcribed spacer (ITS) region. Following the method described by Irinyi (2015), the ITS of the ribosomal DNA (rDNA) region was amplified in a polymerase chain reaction (PCR) using the primers ITS1 (5′-TCCGTAGGTGAACCTGCGG-3′) and ITS4 (5′-TCCTCCGCTTATTGATATGC-3′) (Irinyi et al. 2015). The following parameters were used for the PCR reaction: 98 °C for 30 s, 30 cycles of 98 °C for 10 s, 45 °C for 30 s, 72 °C for 1 min, and 72 °C for 2 min. In addition, the D1/D2 region of the large ribosomal subunit was also amplified and sequenced to provide better discrimination for the yeast isolates. The D1/D2 region was amplified using the primers NL1 (5′-GCATATCAATAAGCGGAGGA-3′) and NL4 (5′-TTGGTCCGTGTTTCAAGACG-3′) following the same PCR parameters as the one used for ITS. The PCR products were evaluated using a 1% gel electrophoresis, then purified using Invitrogen’s PureLink® PCR Purification kit (Thermo Fisher Scientific, Carlsbad, CA, USA). This was followed by quantification using a Nanodrop 2000c (Thermo Scientific, USA).

The PCR amplicons for the ITS and D1/D2 regions were sent to Inqaba Biotech (Pretoria, South Africa). The quality of the sequences was assessed using Snapgene viewer (version 7.0.2). Thereafter, the generated ITS and the D1/D2 consensus sequences were compared to the respective ITS or D1/D2 sequences deposited on the National Centre for Biotechnology Information (NCBI) database using the Basic Local Alignment Search Tool (BLAST) to identify closely related organisms. Phylogenetic trees were constructed to identify the clades these isolates fall within.

### Antifungal susceptibility testing (broth microdilution assay)

The broth microdilution assays were performed following the guidelines provided by the Clinical Laboratory Standard Institute (CLSI) guidelines (CLSI 2017). Briefly, 50 µL of RPMI (Capricorn Scientific, USA) broth was added to all columns from column 2 of a flat bottom 96-well microtiter plate to column 12. Following this, 100 µL of the antifungal stock solution (fluconazole (256 µg/mL, Merck, South Africa), amphotericin B (64 µg/mL, Merck, South Africa), micafungin (128 µg/mL, Merck, South Africa)) was added in column 1. Two-fold serial dilution of the antifungal stock was done by pipetting 50 µL of the antifungal from column 1 to column 11. Freshly prepared fungal culture inoculum standardized to 0.5 McFarland (1.5 × 10^8^ colony forming units, (CFU/ml)) was added to all the wells. The 96-well microtiter plates were then sealed and incubated at 37 °C for 24 h. Column 12 was used as a sterility control; therefore, no antifungal was added. After the incubation period, 10 µL of 0.02% resazurin (Sigma-Aldrich, South Africa) was added to all wells followed by incubation at 37 °C for 2 h. There are currently no established *C. auris*-specific susceptibility breakpoints; therefore, breakpoints commonly used are defined based on those established for closely related *Candida* species. The breakpoints for fluconazole, amphotericin B, and micafungin were ≥ 32 µg/mL, ≥ 2 µg/mL, and ≥ 4 µg/mL, respectively. Fungal growth was indicated by the pink colour while a blue colour indicated the inhibition of fungal growth. The MIC was recorded as the lowest concentration at which the blue colour of the resazurin was maintained.

### Characterization of virulence factors

#### Extracellular enzyme (phospholipase and proteinase) production

##### Phospholipase assay

Phospholipase production was determined as described by Erum et al. (2020). Briefly,10 µL of yeast suspension was inoculated into Sabouraud’s dextrose agar (SDA, Neogen, UK) supplemented with 58.4 g/L NaCl (Sigma-Aldrich, USA), 5.5 g/L CaCl_2_ (Sigma-Aldrich, USA), and 10% egg yolk emulsion (Neogen, USA) and incubated at 37 °C for 72 h. *Candida parapsilosis* ATCC 22019 and *Candida albicans* ATCC 90028 were used as phospholipase production controls (Erum et al. 2020). The production of phospholipase activity (demonstrated by the formation of a clearance zone around the yeast colony) was measured after incubation and interpreted as follows:

- Pz value = [colony diameter in mm][colony diameter + clearance zone]

The Pz values were interpreted as follows:

- Pz = 1.00 indicates a negative phospholipase activity
- Pz < 0.63 shows that the test strain produces a strong amount of phospholipase.
- Pz values ranging from 0.64 to 0.99 imply that the test strain is releasing a weak amount of phospholipase.

##### Proteinase assay

The proteinase activity assay was performed as described by Yousuf et al. (2011) with minor modifications. Briefly, 10 μL of yeast culture suspension containing 1 × 10^8^ CFU/mL was spotted in BSA agar (20 g/L glucose (Sigma-Aldrich, Mexico), 2 g/L bovine serum albumin (Sigma-Aldrich, USA), 1.45 g/L Yeast Nitrogen Base (YNB, Sigma-Aldrich, USA), and 1.45 g/L ammonium sulphate (Sigma-Aldrich, Germany). *Candida parapsilosis* ATCC 22019 and *Candida albicans* ATCC 90028 were used as controls for proteinase production. This was followed by incubating the plates at 37 °C for 72 h. After incubation, the width of the precipitation zone was measured. The proteinase activity was identified as a formation of a whitish zone surrounding each colony. The (Pz) value was calculated as the diameter of the colony/ the diameter of the colony plus the precipitation zone. These Pz values were then interpreted as described in the phospholipase activity (Yousuf et al. 2011).

## Adherence to silicone elastomers

Test for the ability of *C. auris* to adhere to surfaces was done as described by Mateus et al. (2004). Yeast cells were cultured in 10 mL YNB (yeast nitrogen base w/t amino acids) broth (Sigma-Aldrich, USA) followed by overnight incubation at 37 °C. After that, the cells were rinsed twice with a solution of Hanks’ buffered salt (Capricorn Scientific, USA), adjusted to 5 × 10^3^ cells/mL and diluted with the same buffer to achieve a cell suspension of 250 cells/mL. Thereafter, 1.2-cm-diameter silicone elastomer discs (Invitrogen, SA) were placed in each well of the 6-well culture plates, and 2 mL of the yeast cell suspension (yeast cells cultured in 10 mL YNB) was added. The plates were then incubated at 37 °C for 30 min to allow the cells to attach to the elastomer discs. After incubation, discs containing attached cells were transferred to a new 6-well plate with 2 mL of Hanks’ buffered salt solution. After aspiration of the buffer, the discs were rinsed twice with Hanks’ buffered salt solution and overlaid with 3 mL of half-strength Sabouraud’s dextrose agar (Neogen culture media, UK). After incubating the plates at 37 °C for 18 to 24 h, the number of *C. auris* cells attached to the catheter material was counted (Mateus et al. 2004).

## Biofilm formation

*Candida*’s propensity to build biofilms has been connected to catheter-related infections. Here, we used a method described by Larkin et al. (2017) to test for the ability of *C*. *auris* to form biofilms. Silicone elastomers were employed as a biofilm substrate in this study because catheters are typically made of this material. Silicone elastomer (1.2-cm diameter) discs were placed in 6-well tissue culture plates containing foetal bovine serum (Sigma-Aldrich, USA) and shaken for 24 h on a rocker at 37 °C. The discs were then withdrawn from the foetal bovine serum and placed in a 4 mL cell suspension containing 1 × 10^7^ cells/mL and were incubated for 90 min at 37 °C (adhesion phase) to facilitate adhesion. After that, the discs were placed in a 4 mL YNB solution and grown for 24 h at 37 °C to produce mature biofilms (mature phase). The biofilm-covered discs were then transferred to 6-well tissue culture plates containing a mixture of 2,3-bis-(2-methoxy-4-nitro-5-sulfophenyl)-2H-tetrazolium-5-carboxanilide (XTT; 12.5 g/mL; Thermo Scientific, USA), 1 M menadione (Sigma-Aldrich, USA) in phosphate-buffered saline (Invitrogen, SA), and incubated at 37 °C for 3 h to assess metabolic activity. The formation of biofilms was seen by the appearance of a blue to purple colour on the silicone elastomers after a 3-h incubation period (Larkin et al. 2017).

### Characterization of environmental factors

#### Thermotolerance and halotolerance

##### Thermotolerance

Thermotolerance was assessed by sub-culturing *C. auris* isolates and *Candida* controls onto SDA plates followed by observing the growth rate after 24 h of incubation at 42 °C. The isolates that showed growth after incubation for 24 h were marked as thermotolerant.

##### Halotolerance at 42 °C

The ability of *C. auris* to survive under high salinity environments was assessed at higher temperatures by sub-culturing *C. auris* isolates and the *Candida* controls onto SDA plates with 10% NaCl. The isolates that showed growth after 24 h of incubation were marked as halotolerant and thermotolerant.

## Results

### *Candida* auris isolation and identification

#### Identification by sequencing the internal transcribed spacer (ITS) region of the rDNA

**Fig. 1:**
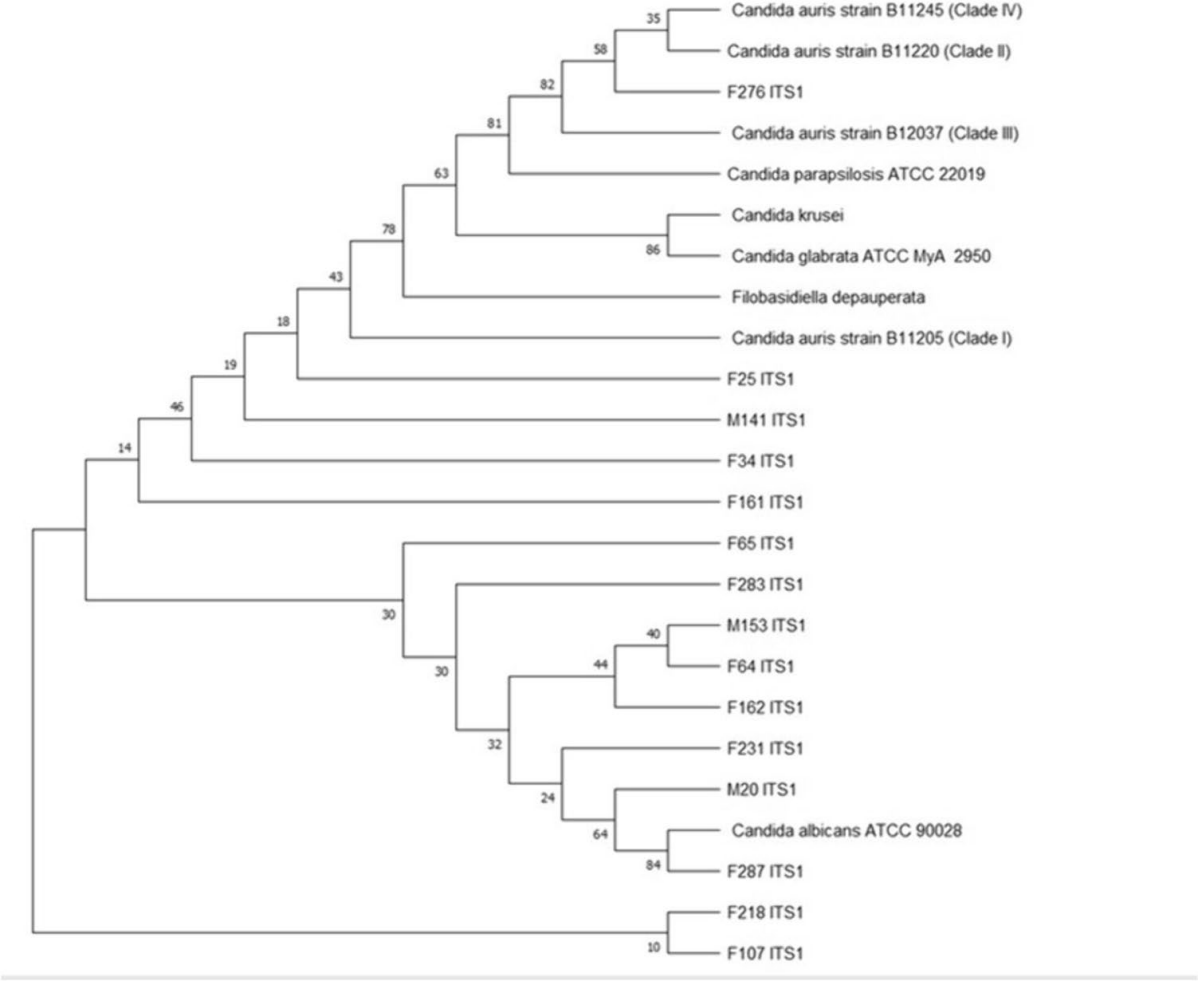
The maximum-likelihood tree based on the ITS sequences of *Candida auris* isolates, with strains from the four *Candida auris* clades accessed from GenBank using the BLASTN tool. The phylogenetic tree was constructed using the software MEGA 11, and the sequences were aligned using ClustalW. Bootstrap values of 1000 replicates are displayed on the branches of the tree

**Fig. 2:**
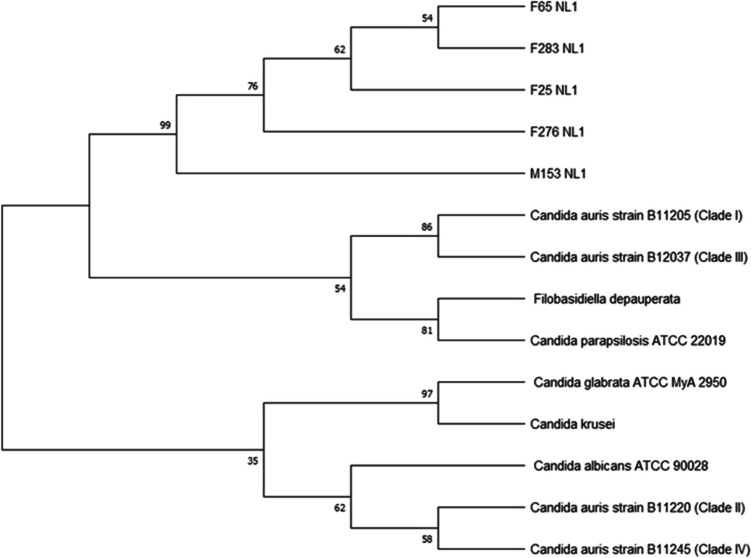
The maximum-likelihood tree based on the D1/D2 sequences of *Candida auris* isolates, with strains from the four *Candida auris* clades accessed from GenBank using the BLASTN tool. The phylogenetic tree was constructed using the software MEGA 11, and the sequences were aligned using ClustalW. Bootstrap values of 1000 replicates are displayed on the branches of the tree

Fifteen putative *Candida auris* isolates were identified by sequencing the internal transcribed spacer (ITS) region. The ITS amplicons were between 250 and 500 bp (Fig. S1, Appendix A) and the sequence analysis of the ITS amplicons showed a similarity percentage of 97–100% (Table 1). Five *C. auris* isolates which were selected by their multi-drug resistance profile were further confirmed by sequencing the D1/D2 region of the large ribosomal subunit of the 28S ribosomal RNA gene using primers specific for yeasts. The D1/D2 amplicons were located at 620 bp (Fig. S2, Appendix A), and the D1/D2 sequence analysis of these amplicons showed percentage identity ranging from 99 to 100% (Table 2). Most of the *Candida auris* isolates sequenced by both the ITS region (Fig. 1) and the D1/D2 region (Fig. 2) showed a close relationship to the *Candida auris* strain B11205, which is from Clade I, thus suggesting that these isolates may be from Clade I.

**Table 1:** Identification of *Candida* isolates based on the internal transcribed spacer (ITS) sequence using nucleotide BLAST search

*Candida* species ID	BLAST hit	Query cover (%)	Percentage identity (%)	Accession no.
*C. albicans* ATCC 90028	*C. albicans*	87	99.69	
*C. parapsilosis* ATCC 22019	*C. parapsilosis*	79	97.66	
F25	*C. auris*	86	100	OR786019
F34	*C. auris*	88	99.68	OR786028
F64	*C. auris*	86	100	OR786017
F65	*C. auris*	90	99.05	OR786015
F107	*C. auris*	80	98.20	OR7860026
F161	*C. auris*	92%	97.73	OR786021
F216	*C. auris*	85	99.04	OR786025
F231	*C. auris*	80	98.20	OR786023
F232	*C. auris*	81	99	OR791934
F276	*C. auris*	100	100	OR789580
F283	*C. auris*	97	100	OR786013
F287	*C. auris*	87	99.69	OR786003
M20	*C. auris*	90	100	OR786005
M141	*C. auris*	86	100	OR786009
M153	*C. auris*	88	100	OR786007

**Table 2:** Identification of *Candida* isolates based on the D1/D2 region of the large subunit of the *28S ribosomal RNA* gene sequences

*Candida* species ID	Scientific name	Query cover (%)	Percentage identity (%)	Accession no.
*C. albicans* ATCC 90028	*C. albicans*	100	100	
*C. parapsilosis* ATCC 22019	*C. parapsilosis*	100	99.81	
F25	*C. auris*	100	100	OR789339
F65	*C. auris*	99	99.81	OR789337
F276	*C. auris*	100	100	OR789341
F283	*C. auris*	97	100	OR789343
M153	*C. auris*	100	100	OR789345

### Antifungal susceptibility testing (broth microdilution assay)

Fifteen *C. auris* isolates were tested for antifungal susceptibility against three selected antifungal compounds, fluconazole, amphotericin B, and micafungin, representing the azoles, polyenes, and echinocandin classes, respectively (Table 3). Out of these 15 tested isolates, 10 were resistant to fluconazole, 8 were resistant to amphotericin B, and 5 were resistant to micafungin. Five *C. auris* isolates (*C. auris* F25, *C. auris* F65, *C. auris* F283, *C. auris* F276, and *C. auris* M153) showed resistance to all three selected antifungals (fluconazole, amphotericin B, and micafungin), and were marked multidrug resistant. The negative control showed no activity suggesting that the solvent used to dissolve fluconazole and micafungin did not contribute to the observed activity profile.

**Table 3:** Antifungal susceptibility patterns of *Candida auris* isolates against three major antifungal groups (azoles, polyenes, and echinocandins). All tests were done in duplicates, and the results are reported as the average of the two MICs

*Candida* species ID	Minimum inhibitory concentration (µg/mL)		
	Fluconazole	Amphotericin B	Micafungin
*C. albicans* ATCC 90028	0.5	0.25	0.1565
*C. parapsilosis* ATCC 22019	2	1.5	2
*C. auris* F25	48	32	8
*C. auris* F34	24	8	3
*C. auris* isolate F64	64	0.1875	0.5
*C. auris* isolate F65	48	8	8
*C. auris* F107	16	1	3
*C. auris* F161	32	1.5	2
*C. auris* F216	24	1	0.75
*C. auris* F231	8	1.5	0.3125
*C. auris* 232	12	0.5625	0.1875
*C. auris* F276	48	24	8
*C. auris* F283	48	64	16
*C. auris* F34	24	8	3
*C. auris* F287	32	4.5	0.5626
*C. auris* M20	96	1.5	2
*C. auris* M141	64	3	3
*C. auris* M153	48	12	4

Tentative breakpoints as classified by CDC: Fluconazole (Resistant ≥ 32 µg/mL, Susceptible < 32 µg/mL), Amphotericin B (Resistant ≥ 2, Susceptible < 2), and Micafungin (Resistant ≥ 4, Susceptible < 4)

### Characterization of adaptive features

#### The ability of C. auris isolates to survive in high temperatures (42 °C) and high salinity (10% sodium chloride)

Five *C. auris* isolates were further tested for their ability to withstand high temperatures and salinity. All five tested *C. auris* isolates were both thermotolerant (42 °C) and halotolerant (10% NaCl; Table 4).

**Table 4:** Thermotolerance (42 °C) and halotolerance (10% sodium chloride) patterns presented by selected multidrug-resistant *C. auris* isolates

*Candida* species ID	Thermotolerance (42 °C)
*C. albicans* ATCC 90028	-
*C. parapsilosis* ATCC 22019	+
*C. auris* F25	+ + +
*C. auris* F65	+ +
*C. auris* F276	+ + +
*C. auris* F283	+ + +
*C. auris* M153	+ + +

(-), no growth; ( +), poor growth; (+ +), moderate growth; (+ + +), high growth

#### Production of extracellular enzymes (phospholipase and proteinase)

Five *C. auris* isolates showing resistance to all antifungals used in this study were tested for their ability to produce extracellular enzymes (phospholipase and proteinase) under different temperature conditions (37 °C and 42 °C, Table 5). All the *C. auris* isolates produced phospholipase and proteinase at 37 °C. At 42 °C, all the isolates produced proteinase, but none produced phospholipase.

**Table 5:** Production of extracellular enzymes (phospholipase and proteinase) by selected multidrug-resistant *Candida auris* isolates under different temperature conditions (37 °C and 42 °C)

*Candida* species ID	Phospholipase activity		Proteinase activity	
	37 °C	42 °C	37 °C	42 °C
*C. albicans* ATCC 90028	0.57	-	0.21	-
*C. parapsilosis* ATCC 22019	0.46	-	0.27	-
*C. auris* F25	0.47	-	0.24	0.50
*C. auris* F65	0.56	-	0.24	0.57
*C. auris* F276	0.47	-	0.31	0.55
*C. auris* F283	0.52	-	0.34	0.63
*C. auris* M153	0.45	-	0.27	0.66

Pz < 0.63, very strong enzyme production; Pz between 0.64 and 0.99, weak enzyme production; Pz = 1, very weak enzyme production; (-), inability to produce enzyme

#### The ability of C. auris to form biofilms and adhere to elastomer discs

The selected *C. auris* isolates were further tested for their ability to form biofilms and adhere to silicone elastomers. All the tested isolates formed biofilms and also adhered to the elastomers (Table 6).

**Table 6:** Adherence activity and biofilm formation of *C. auris*

*Candida* species ID	Number of cells adhered	Biofilm formation
*C. albicans* ATCC 90028	-	-
*C. parapsilosis* ATCC 22019	302	+ +
*C. auris* F25	528	+ + +
*C. auris* F65	492	+ + +
*C. auris* F276	356	+ + +
*C. auris* F283	372	+ + +
*C. auris* M153	408	+ + +

(-), inability to adhere and form biofilms; (+ +), strong biofilm formation ability; (+ + +), very strong biofilm formation ability

## Discussion

In this study, fifteen putative *C. auris* isolates were identified using the ITS region of the rDNA as the primary target region. The sequences obtained from ITS amplicons with base pairs between 500 and 1000 were analyzed using the BLAST search from NCBI. The BLAST search of the ITS sequences showed sequence identities ranging from 97 to 100% (Table 1). The control *Candida* strains were also accurately identified using the ITS region, indicating confidence in using the ITS region for the identification of the putative *C. auris* isolates. Molecular methods based on the ITS rDNA region and mass spectrometry–based methods such as MALDI TOF MS are regarded as the gold standard methods for the accurate identification of *C. auris* (Fasciana et al. 2020). The challenge, even for highly resourced laboratories, is that MALDI TOF MS is a high-end technique that is not readily available in most laboratories. On the other hand, sequencing is becoming less expensive and within the reach of individual laboratories with the introduction of pocket-sized sequencing machines (Haider et al. 2023). Therefore, ITS sequencing might be a practical tool for quick identification or confirmation of *C. auris* isolates even though this technique might be a challenge in small diagnostic laboratories in lower to middle-income countries because of the cost and availability of molecular expertise. Identification of *C. auris* using commonly available diagnostic methods in laboratories can be challenging with standard laboratory methods and often leads to misidentification as closely related species such as *C. haemulonii*, *C. famata*, and *C. lusitaniae* (Hata et al. 2019).

The ITS region was chosen by the consortium of mycologists as the standard barcode for fungal species (Raja et al. 2007). This region evolves the fastest, resulting in high variation thus allowing for differentiation among closely related species (Raja et al. 2017). Additionally, to the ITS region, the LSU region which includes the D1/D2 region can provide valuable information for species-level identification in fungi (Ceballos-Escalera et al. 2022). Here, we also performed amplification of the D1/D2 of the ribosomal DNA of selected multidrug-resistant *C. auris* isolates. Following sequencing of the D1/D2 region amplicons with 620 bp, all five multidrug-resistant isolates were confirmed to be *C. auris.* These two domains (ITS region and D1/D2) are highly prevalent in fungal taxonomy and systematics and are recommended for identifying species in natural product research (Raja et al. 2007). While these regions may provide accurate identification of emerging fungal species like *C. auris* and inform decisions on diagnosis and treatment, more research is required to develop rapid and sequencing-free identification methods that can be accessible even in poorly resourced settings.

The minimum inhibitory concentrations (MICs) for the three antifungal compounds were tested against 15 *C. auris* isolates and control *Candida* strains. The result from this study indicates that ten of the fifteen tested *C. auris* isolates were resistant to fluconazole (MIC ranging from 32 to 96 µg/mL), eight were resistant to amphotericin B (MIC ranging from 3 to 64 µg/mL), and five isolates were resistant to micafungin (MIC ranging from 4 to 16 µg/mL; Table 2). The resistance patterns observed in this study are similar to the generally observed *C. auris* resistance patterns where they show intrinsic resistance to azoles, variable resistance to polyenes, and less resistance to echinocandins (Deshkar et al. 2022; Kean and Ramage 2019). For the two *Candida* controls, both *C. parapsilosis* ATCC 22019 and *C. albicans* ATCC 90028 showed susceptibility to all the antifungal classes within the ranges recorded in literature (CLSI 2017). The antifungal resistance of *Candida auris* isolates depends on the clade to which the species belongs. Nevertheless, these results emphasize the recently observed antifungal resistance patterns where non-*albican Candida* species are emerging as the most resistant fungal pathogens compared to a well-known fungal pathogen, *C. albicans* (Deorukhkar et al. 2014).

The most disturbing issue with *C. auris* is its multidrug resistance profile (Eliaš and Gbelská 2022). In this study, five *C. auris* isolates showed resistance to all three antifungal drug classes. The resistance profiles of these isolates raise questions on their evolution, ability to invade human hosts, ability to cause infections, and spread from patient to patient. The consensus is that *C. auris* was a non-pathogenic environmental species that adapted to mammalian temperatures through evolution. The earliest evidence of *C. auris* as a human pathogen dates from 1996, identified through a retrospective study of an unidentified *Candida* isolate from a bloodstream infection in a South Korean child with hypoxic encephalopathy and aspiration pneumonia (Lee et al. 2011). Before that, there was no evidence that *C. auris* was already a human pathogen (Casadevall et al. 2019). The transition of a fungal species from a non-pathogen to an infectious pathogen of humans is facilitated by several pathogenic attributes, including virulence factors such as adherence to host tissues, biofilm formation, and production of extracellular enzymes for host penetration, and other adaptive features such as resilience at high salinity and temperature (Deorukhkar et al. 2014). Indeed, some of the genes encoding these virulence factors in *C. albicans* have been identified from the genome of *C. auris* (Rossato and Colombo 2018). We assumed that the isolates showing multidrug resistance patterns also display interesting pathogenic attributes and adaptive features that assist these specific functions like host penetration (secretion of extracellular enzymes), resilience to harsh conditions like the skin and human body temperature, adhering to surfaces, and spreading from patient to patient. We then selected all five *C. auris* isolates displaying multidrug resistance for further characterization of these attributes.

Multidrug-resistant *C. auris* isolates have been characterized to exhibit tolerance to higher temperatures and harsh environments, which are fitness factors believed to have helped this pathogen adapt to new ecological niches such as mammalian host skin (Sun et al. 2020). We assessed the ability of the five selected isolates to tolerate higher temperatures (42 °C). We observed that all *C. auris* isolates were able to maintain moderate to good growth patterns even at higher temperatures. The *Candida* control (*C. albicans*) however was unable to grow at this elevated temperature, while *C. parapsilosis* also showed poor growth. These results suggest that this species is better adapted to high temperatures compared to the species in the same family. These findings are consistent with the thermotolerance analysis performed on *C. auris*, which suggested that the organism may have become acclimated to the rise in ambient temperatures brought on by global warming to adapt to and survive at various host temperatures (Jackson et al. 2019; Casadevall et al. 2019; Di Luca et al. 2019).

Since *C. auris* isolates have been shown to tolerate both higher temperatures and salinity (10% NaCl), we further screened these isolates for growth under the combination of these parameters. All *C. auris* isolates maintained their growth patterns under both selection pressures (42 °C and 10% salinity). It was interesting to note that *C. parapsilosis* lost its growth ability when cultured under both thermal and salinity stress. *C. parapsilosis* is known to grow at temperatures ranging from 20 to 42 °C; however, it was previously observed that *C. parapsilosis* growth was inhibited at 42 °C in the presence of sodium chloride (Silva et al. 2012; Aryal 2022). It was proposed that high temperatures and salinity may cause changes in the fungus’s cell membrane and cell wall, resulting in its inability to grow under these conditions (Ene et al. 2012). Recently, *C. auris* has been isolated in high salt marsh, suggesting that they evolved the ability to adapt to warm and highly saline environments. The ability of *C. auris.* to survive under high temperatures and high salinity facilitates its prolonged survival in the human body, thereby increasing the course of infection. However, the mechanism by which *C. auris* can adapt and thrive under these stress combinations is yet to be elucidated.

We further assessed the ability of the selected multidrug-resistant *C. auris* isolates to produce extracellular enzymes (phospholipase and proteinase), which might help this pathogen invade mammalian host tissues in the new ecological space. Here, we observed that all the tested *C. auris* isolates had very strong production (*P*_z_ < 0.63 as stated by Larkin et al. 2017) of both phospholipase and proteinase at 37 °C, comparable to that of *C. albicans* (P_z_ = 0.57, P_z_ = 0.21 for phospholipase and proteinase, respectively), which is known to be the best producer of these extracellular enzymes. Even though both the enzymes were produced strongly, phospholipase was produced at a lower rate than proteinase. Several studies that assessed the ability of *C. auris* to produce extracellular enzymes (phospholipase and proteinase) reported that proteinase production was higher than that of phospholipase (Larkin et al. 2017; Arikan-Akdagli et al. 2018; Shaban et al. 2020). The reason behind the production of more proteinase than phospholipase by *C. auris* is unknown. However, this finding could imply that proteinases are the most important contributors to *Candida* pathogenicity, since they are involved in several functions such as host tissue destruction, cell wall formation, and host cell evasion (Watkins et al. 2022).

Since multidrug-resistant *C. auris* isolates can grow at elevated temperatures and high salinity, we found it interesting to evaluate the stability of the produced extracellular enzymes at a higher temperature of 42 °C. We observed that all the *C. auris* isolates lost the ability to produce phospholipase at 42 °C, but they were able to maintain proteinase production at this temperature, albeit at reduced levels compared to the production at 37 °C. Proteinase enzymes secreted by *C. auris* break down proteins and are important for the survival of *C. auris* in the host environment (Rossato and Colombo 2018). Therefore, this suggests that *C. auris* produces proteinase enzymes at 42 °C to help it better adapt to higher temperatures so that it can be able to survive in the human host (Watkins et al. 2022). However, more research is required to comprehend the underlying mechanism behind the production of only proteinase enzymes at elevated temperatures.

One of the adaptive features of *C. auris* is the ability to adhere to surfaces and persist in hospital environments and medical devices, thus leading to the spread of this fungal pathogen from patient to patient (Rossato et al. 2022). As a result, *C. auris* has been linked to several outbreaks around the world, including in the UK, Spain, Norway, and Germany from April 2015 to November 2016, as well as in South Africa around June 2019 (Eyre et al. 2018; Borman and Johnson 2020). Here, we assessed the ability of the selected multidrug-resistant *C. auris* isolates to adhere to surfaces and to form biofilms. We used silicone elastomers to simulate hospital catheters which are typically made from this material. The ability to form biofilms and adhere to elastomers was observed in all the multidrug-resistant *C. auris* isolates tested in this study. The *C. auris* isolates produced a significant number of biofilms and adherent cells as compared to *C. parapsilosis* which showed moderate biofilm production and a lower number of adherent cells. *Candida albicans* were unable to form biofilms nor adhere to the silicone elastomers even though they are known to form true biofilms and adhere to surfaces. *Candida albicans*’ inability to adhere and form biofilms could be explained by the fact that the formation of biofilms in *Candida* species is strain-dependent (Cavalheiro and Teixeira 2018; Pereira et al. 2021).

Thus, these findings show that *C. auris* has a greater adherence to elastomers and biofilm formation than *C. parapsilosis* and *C. albicans*, which are known to cause catheter-related candidiasis. The ability of *Candida* species to adhere to catheter surfaces is one of the key factors responsible for catheter-associated candidiasis (Ahmad and Alfouzan 2021). Therefore, the strong ability of *C. auris* to adhere to elastomers as seen in this research suggests that this pathogen plays a significant role in causing catheter-related candidiasis whereas its ability to form biofilms aids the pathogen’s prolonged survival on surfaces, resulting in its spread within healthcare settings (Ahmad and Alfouzan 2021). All the isolates that formed biofilms also showed the ability to adhere to elastomer discs. Therefore, this could imply that adherence and biofilm formation are co-dependent; thus, for the pathogen or microbe to form biofilms, they must first adhere to surfaces. These two traits may facilitate the transmission of *C. auris* infections in hospital environments.

## Conclusion

In the current study, we assessed the antifungal susceptibility patterns of *Candida auris* when treated with amphotericin B, micafungin, and fluconazole. The majority of the tested *C. auris* isolates showed heightened resistance to fluconazole, with variable susceptibility to micafungin and amphotericin B. This study discovered that some isolates of *C. auris* exhibited a multidrug resistance profile, which is a serious challenge for the global healthcare system considering the paucity of effective antifungals. We also observed that *C. auris* produces proteinase at higher temperatures, suggesting that this enzyme aids this pathogen in its ability to colonize and destroy host tissues and also make it adaptable for survival in the mammalian body. *C. auris* utilizes these factors to gain entry and survive in the human body, to facilitate its persistence on both the skin and hospital surfaces. The ability of *C. auris* to penetrate the host, its multidrug resistance profile, and its ability to tolerate high temperatures and salinity confirms that it is a health threat that needs immediate intervention. Furthermore, the findings of this study emphasize the importance of researching and developing new treatment strategies and antifungal drugs to effectively treat and control invasive fungal infections brought on by emerging fungal pathogens like *C. auris*.

## Supplementary Information

Below is the link to the electronic supplementary material.

Supplementary file1 (DOCX 1093 kb)

## Data Availability

No datasets were generated or analysed during the current study.
