# Supplementary material for: Characterization of susceptibility patterns and adaptability of the newly emerged Candida auris
Source: Int Microbiol. 2024 Aug 7;28(3):575–87. doi: 10.1007/s10123-024-00563-1 (PMC11906518; doi:10.1007/s10123-024-00563-1)
Supplement: Supplementary file 1 — Supplementary file1 (DOCX 1093 kb) [file 10123_2024_563_MOESM1_ESM.docx]

**Supplementary Material**


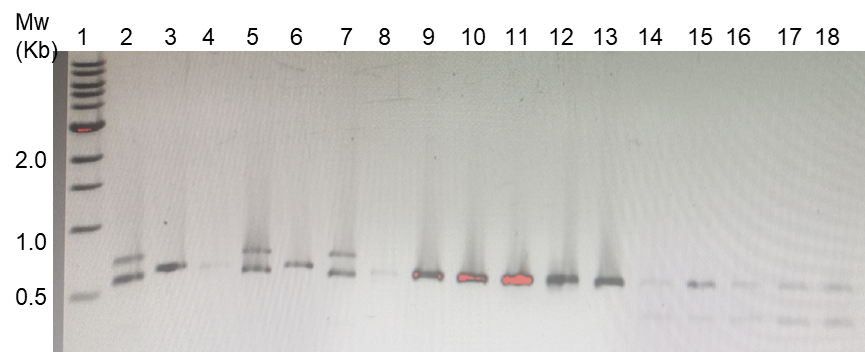


**Figure S1:** Amplicons of the internal transcribed spacer region of the *C. auris* rDNA. Lane 1:500 bp Molecular weight marker, Lane 2: *C. albicans* ATCC 90028, Lane 3-18: putative *C. auris* isolates (F25, F34, F64, F65, F107, F161, F216, F231, F232 F276, F283, F287, M20, M141, M153).


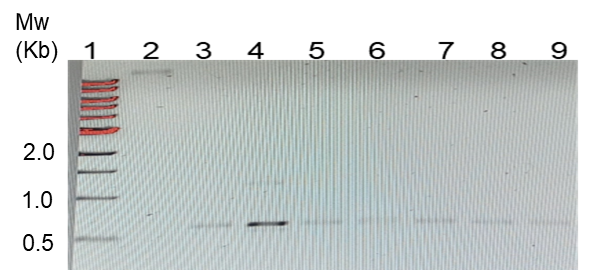


**Figure S2:** Amplicons of the D1/D2 region of the large ribosomal subunit of the *28S ribosomal RNA* gene. Lane 1: 1 kb molecular weight marker, Lane 2: Control (16Mb *C. albicans* gDNA), Lane 3-5: ATCC *Candida* controls (*C. albicans* ATCC 90028, *C. glabrata* ATCC MYA 2950, and *C. parapsilosis* ATCC 22019), Lane 7-9: *Candida auris* isolates (F25, F65, F276, F283 and M153).
